# The LIQUID endstation for *in situ* spectro-electrochemical studies on energy materials @ BACH beamline

**DOI:** 10.1107/S1600577526005825

**Published:** 2026-07-15

**Authors:** I. Napal Azcona, F. Salvador, Z. Liang, G. Cherkashinin, M. Sosič, D. Benedetti, E. Betz-Güttner, S. Dal Zilio, A. Fondacaro, F. Bondino, I. Píš, M. Lazzarino, S. Nappini, E. Magnano

**Affiliations:** a CNR – Istituto Officina dei Materiali (IOM) Basovizza Area Science Park 34149Trieste Italy; b Physics Department Università degli Studi di Trieste P. le Europa 1 34127Trieste Italy; c Institute of Materials Sciences Technical University Darmstadt 64287Darmstadt Germany; d Institute of Electrical Engineering Slovak Academy of Sciences Dubravska cesta 9 84104Bratislava Slovakia; NSRRC, Taiwan

**Keywords:** soft X-ray absorption spectroscopy, electro-chemical microfluidic cell, *operando*, batteries

## Abstract

A new endstation completely dedicated to the study of X-ray absorption spectroscopy in the soft X-ray energy range in liquid environments has been developed and implemented on one of the three branch lines of BACH beamline at Elettra Sincrotrone Trieste. The new endstation can host different cells developed for various purposes and is available to researchers for experiments. The first prototype of cell developed in 2018 has been upgraded and made compatible with the new endstation. Alongside this, a second cell dedicated to experiments with materials for batteries has been implemented. Here we report all the technical specifications of the new apparatus along with examples of demonstrated applications of the cells.

## Abstract 2

A new endstation for soft X-ray absorption spectroscopy in liquid environments has been installed at the BACH beamline of Elettra Sincrotrone Trieste. The setup includes upgraded and newly developed cells for a range of soft-X-ray experiments at solid/liquid interfaces, including studies on battery materials.

## 1. Introduction

Soft X-ray absorption spectroscopy (soft-XAS) has established itself as a powerful technique for investigating the local electronic and structural properties of matter with elemental and orbital specificity. Its sensitivity to oxidation states, unoccupied density of states, and chemical bonding make it indispensable for studying functional materials, catalysts, and electrochemical interfaces.

Traditionally, soft-XAS has been performed under ultra-high-vacuum (UHV) conditions, which has limited its application to solid samples and prevented the direct investigation of systems operating in liquid environments. However, many of the most pressing scientific questions, which range from the mechanisms of electrocatalytic reactions to the charge–discharge processes in energy storage devices, require probing materials under realistic operating conditions, where liquid electrolytes and applied potentials are essential components of the system.

In recent years, significant progress has been made in bridging this gap, with the development of specialized electrochemical cells and flow devices compatible with synchrotron-based soft X-ray measurements. These setups rely on thin membranes, typically Si_3_N_4_ or related materials, which separate the liquid environment from the UHV chamber while allowing X-rays to penetrate the catalyst/electrolyte interface (Velasco-Velez *et al.*, 2014 ▸; Liu *et al.*, 2015 ▸; Guo *et al.*, 2007 ▸; Fuchs *et al.*, 2008 ▸; Nagasaka *et al.*, 2015 ▸; Kumar *et al.*, 2024 ▸).

New ideas for flow cells have been reported in the literature that improve both reaction control and *operando* characterization. For example, in the cell developed by Tesch *et al.*, the counter electrode (CE) is located in a compartment separated from the working electrode (WE) by a proton conductive membrane to avoid interference of dissolved materials and accumulation of products generated at the CE with the reaction processes occurring at the WE (Tesch *et al.*, 2022 ▸). A different approach was proposed by Mom *et al.* (2019 ▸), who designed a cell where a monolayer of graphene is placed on top of the catalyst, supported by a proton exchange membrane which allows both XAS and X-ray photoelectron spectroscopy (XPS) measurements in the near ambient pressure XPS chamber.

Despite these advances, *operando* soft-XAS measurements remain experimentally demanding. Key challenges include ensuring reliable liquid sealing, maintaining stable catalyst on the membrane, and preventing membrane rupture due to gas evolution or pressure build-up, with stable electrochemical operational conditions during data acquisition. Furthermore, the design of cells must balance mechanical robustness, chemical compatibility, and ease of integration with beamline endstations, which requires custom adaptations for specific experimental geometries.

To address these challenges and provide the research community with a versatile and reliable infrastructure, a new endstation dedicated entirely to soft-XAS in liquid environments has been developed at the BACH beamline of Elettra Sincrotrone Trieste (Zangrando *et al.*, 2004 ▸; Zangrando *et al.*, 2001 ▸). This endstation has been designed to host multiple types of electrochemical cells, tailored to different classes of experiments and research needs. The first prototype of a microfluidic electrochemical cell, originally realized in 2018 (Nappini *et al.*, 2021 ▸), has been upgraded and optimized for integration into the new endstation. This cell enables *operando* studies of the oxygen evolution or reduction reaction (OER, ORR), hydrogen evolution reaction (HER), and CO_2_ reduction (CO_2_RR), providing direct insights into catalytic intermediates and dynamic changes at the electrode–electrolyte interface. In parallel, a second cell has been developed to address the growing demand for *operando* investigations of battery materials. This static configuration allows researchers to follow the evolution of active materials during charge–discharge cycles, offering valuable information on redox processes, phase transformations, and degradation pathways under working conditions.

In the present work, we describe the design and technical specifications of this new endstation and the associated electrochemical cells. Particular emphasis is given to the engineering solutions adopted to ensure operational stability, compatibility with the beamline environment, and flexibility for different experimental demands. Finally, we illustrate the potential applications of the system through representative examples, demonstrating how this infrastructure expands the experimental possibilities for soft X-ray spectroscopy in the study of energy conversion and storage materials.

## 2. Branch-line design and instrumentation

**Figure 1:**
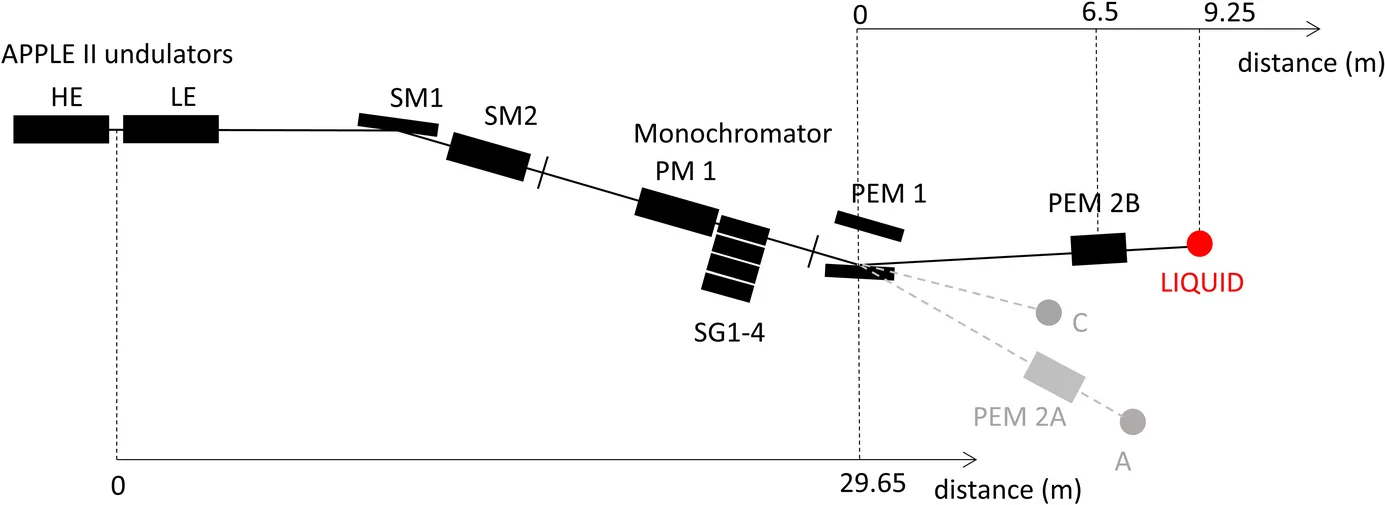
Scheme of the BACH beamline at Elettra Sincrotrone Trieste. The main components of the beamline are highlighted: two APPLE II undulators; prefocusing section: SM1, SM2 spherical mirrors; Padmore variable-angle spherical grating monochromator: PM1 plane mirror, SG1–4: spherical gratings; refocusing section: PEM1, PEM2 plane elliptical mirrors. For more details see Zangrando *et al.* (2001 ▸).

The new endstation dedicated to *operando*/*in situ* experiments (LIQUID) is mounted on one of the three branch lines of BACH beamline (see scheme in Fig. 1 ▸).

Energy tunability and monochromatization are provided by a Padmore variable-angle spherical grating monochromator, comprising a plane mirror and four spherical gratings, and covering the UV to soft X-ray energy range from 44 to 1650 eV. For *operando* measurements the typical energy range used goes from the O *K*-edge (530 eV) to the Al *K*-edge (1570 eV). Under standard operating conditions, with entrance/exit slit openings of 30 µm/30 µm, the energy resolution is approximately 0.15 eV at 500 eV, 0.20 eV at 700 eV, 0.65 eV at 1150 eV, and 1.0 eV at 1500 eV.

**Figure 2:**
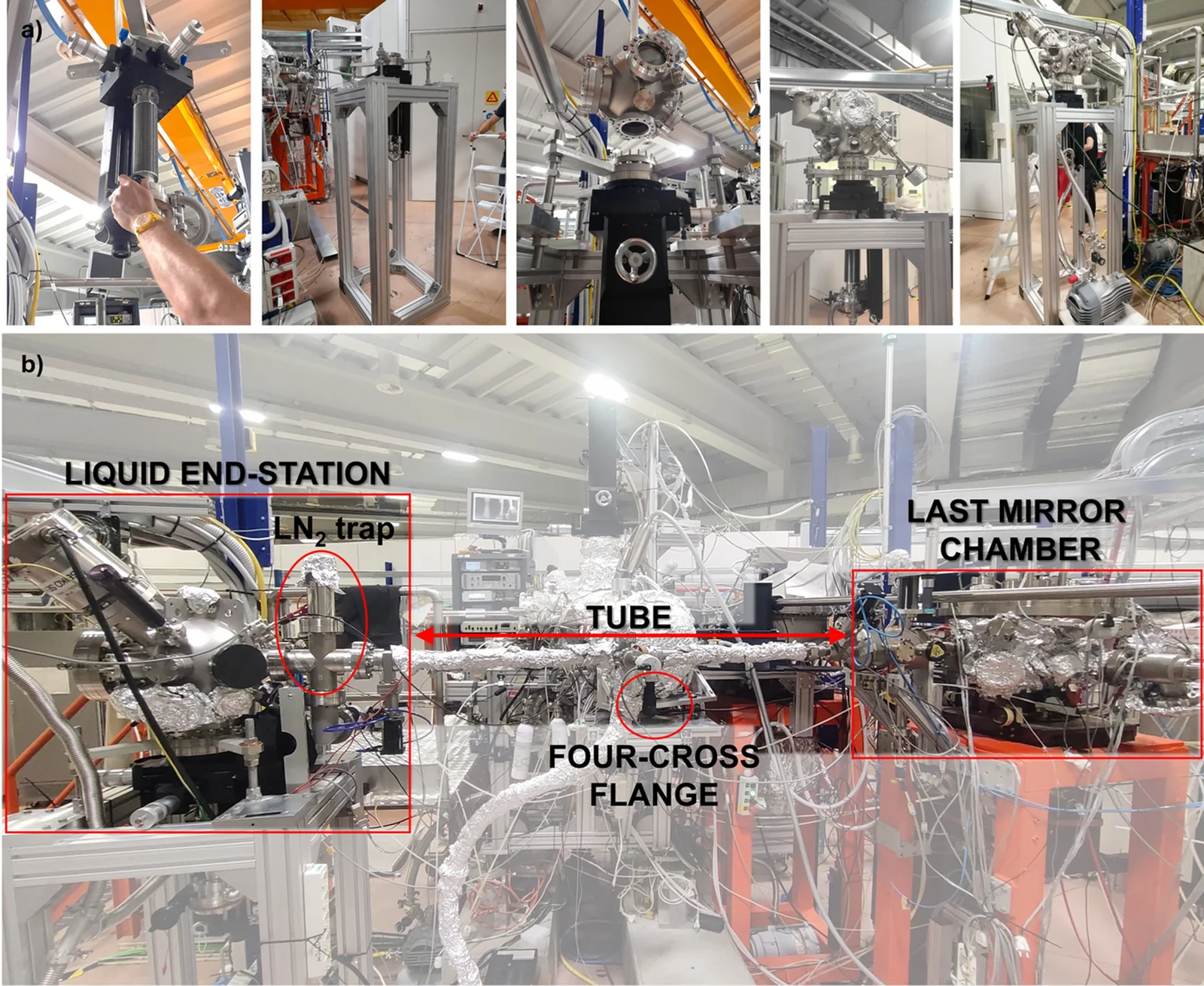
(*a*) Photographs of the assembling works of the LIQUID endstation on BACH beamline. (*b*) Photograph of the final assembly of the branch line with the LIQUID endstation connected to the beamline. Adapted from Napal Azcona (2025 ▸).

**Figure 3:**
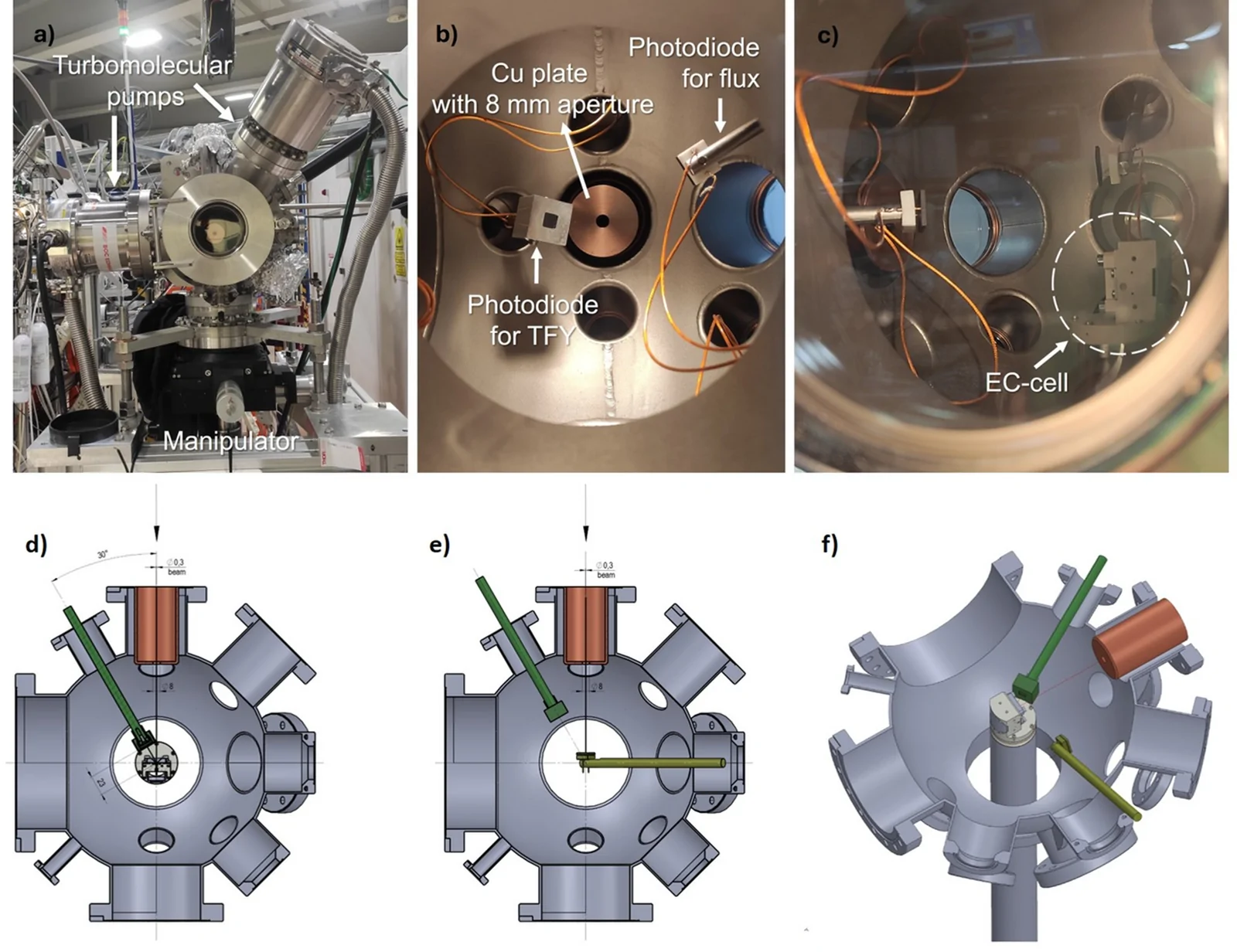
Photograph of the LIQUID endstation: (*a*) two turbomolecular pumps are mounted on top and lateral flanges; (*b*, *c*) internal views of the endstation. The two photodiodes are mounted on linear translators, allowing precise positioning of the measurement photodiode relative to the cell and enabling the flux-monitoring photodiode to be retracted during cell use. Adapted from Napal Azcona (2025 ▸). Technical drawings of the photodiode-beam configurations: (*d*) measurement photodiode in the acquisition position; (*e*) flux-monitoring photodiode in the acquisition position; (*f*) 3D internal view of the endstation.

In Fig. 2 ▸ we report a picture of the mounting steps of the endstation and its final configuration. The endstation is centered at a height of 200 cm from the ground. The tube that connects the last mirror chamber to the endstation is equipped with a four-way-cross-flange where a Si_3_N_4_ membrane can be used to separate the branch line from the rest of the beamline, allowing the vacuum of the beamline to be protected. At the beginning and at the end of the tube, two Cu plates with an aperture of 8 mm are located to preserve a good vacuum in the last mirror chamber. The tube is pumped from the chamber through a by-pass bellow. The endstation is a standard stainless steel (AISI 304L) chamber of 306 mm diameter equipped with 18 flanges and connected to the beamline through a liquid nitro­gen (LN_2_) trap. The chamber is pumped through two 100 l s^−1^ turbomolecular pumps directly mounted on top and lateral flanges of the chamber [see Fig. 3 ▸(*a*)]. The LN_2_ trap has been designed and implemented both to decrease the pressure in the chamber by pumping the residual atmospheric gas (basically H_2_O) and to minimize the potential leakage of liquid towards the beamline, as this could pose a significant risk to the vacuum conditions of the beamline. It consists of a Cu cylinder of 5 cm diameter located at the entry flange of the chamber. The cylinder is closed on both sides by Cu plates featuring 8 mm apertures which allow the entrance of the beam and its horizontally and vertically alignment on the cell. The cylinder is screwed to a dewar which allows cooling of the whole trap. The LN_2_ trap must be refilled approximately every 6 h to maintain the necessary vacuum conditions for the experiment.

### 2.1. Detectors and beam

Two XUV-100 absolute Si photodiodes (IRD AXUV100) are mounted in the chamber on linear translators (see Fig. 3 ▸). The first, used for signal detection during X-ray absorption measurements in fluorescence, is located at 30° from the incident beam, and can be positioned at 2–3 cm from the cell to maximize signal intensity with an acceptance angle of 12.3° at a distance of 2.3 cm, which corresponds to the configuration typically used; the second is movable and can be positioned in line with the beam and used for the calibration of the beam flux intensity or for transmission measurements and retracted when not used.

The signal detection limit is constrained by the photodiode’s low quantum efficiency, which restricts sensitivity to material quantities greater than 1 monolayer (ML) equivalent. For lower amounts, the signal cannot be reliably distinguished from the background noise. The use of photodiodes, despite their low quantum efficiency, is primarily motivated by their significantly lower cost compared with alternative detector technologies. This cost advantage enables a more sustainable and practical approach to maintenance, as it allows for straightforward and economical replacement in the event of failures, such as membrane rupture.

The noise level of *operando* XAS spectra typically falls in the 2–4% range, which is significantly higher than that achievable in *ex situ* measurements (<1%). This increase is primarily due to experimental constraints, including the nature of the sample, such as dilute systems, disordered materials, or species with low absorption cross-sections, which inherently yield weaker signals, and limited acquisition time. Despite these limitations, the data quality remains sufficient to reliably track changes in oxidation states during ongoing reactions.

The photon flux (defined as the number of photons per second), measured at a photon energy of 970 eV at the focal position with entrance and exit slits set at 50 µm, is 2 × 10^11^ photons s^−1^. More information is given in Section S1 of the supporting information. The beam size was evaluated to be 190 µm (V) × 340 µm (H) (FWHM).

#### 2.1.1. Radiation damage: effects and mitigation approaches

Radiation damage represents a significant limitation in soft X-ray investigations of liquids and electrochemical systems, as prolonged exposure can induce chemical alterations, bubble formation, and changes in local electronic structure that compromise data reliability.

Mitigation strategies, including continuous liquid flow to refresh the illuminated volume and beam attenuation to reduce dose, are essential to minimize these artifacts. Evaluating how these approaches influence the reproducibility and stability of the measurements is crucial, as they directly impact the reliability and interpretation of the collected data. A more detailed discussion of these effects is reported by Garcia-Diez *et al.* (2025*a* ▸) and references therein.

Here we provide an example of beam-induced damage which produces a change in the oxidation state of copper-based electrocatalyst, and the specific mitigation strategies applied during experiments. For the synthesis of Cu catalyst, the same procedure as reported in Section S3 was followed.

**Figure 4:**
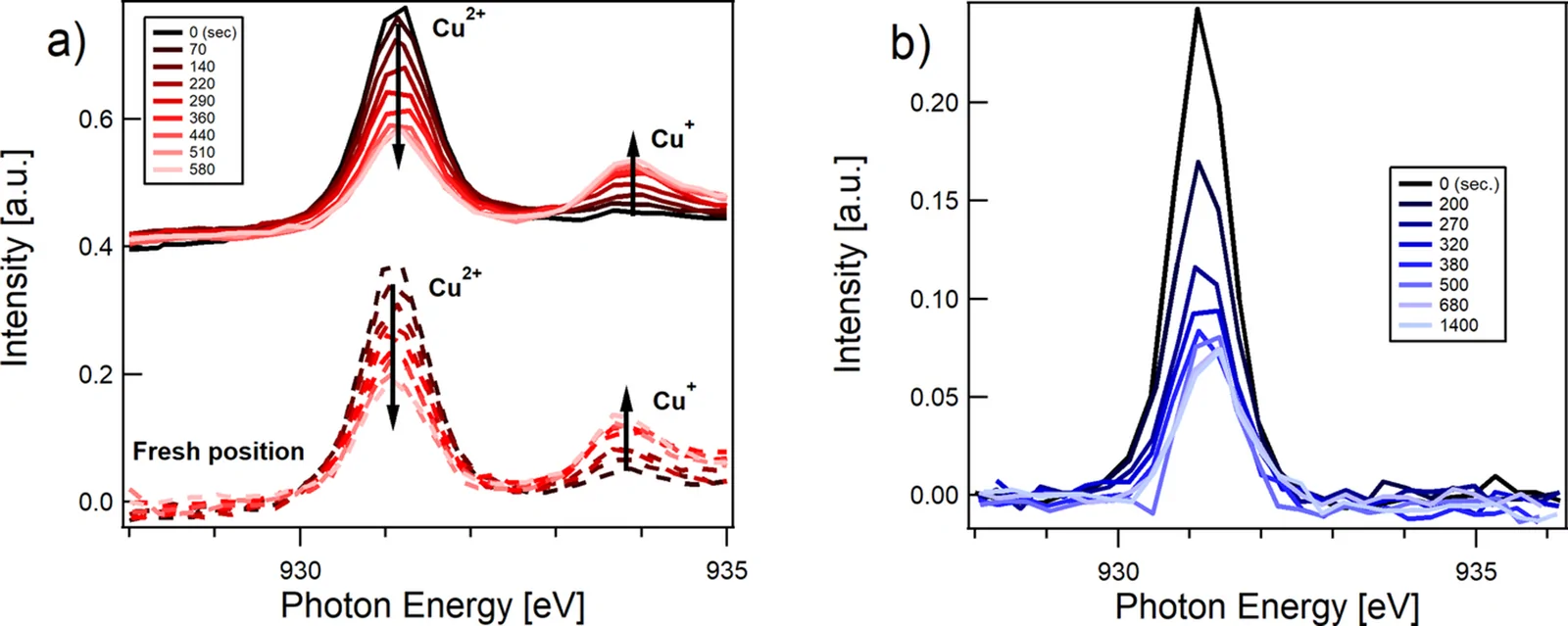
*In situ* Cu *L*_3_-edge TFY-XAS taken repeatedly in an H_2_O environment. (*a*) Beam-induced reduction of Cu oxide species and (*b*) progressive dissolution of Cu catalyst over time. All spectra are shown without any normalization.

The initial *in situ* Cu *L*-edge measurements in an H_2_O environment, shown in Fig. 4 ▸(*a*), revealed a clear beam-damage effect. An important aspect to consider during *in situ* experiments at solid–liquid interfaces is the generation of highly reactive species through water radiolysis upon interaction with the X-ray beam (Carbonio *et al.*, 2020 ▸; Nagy & You, 1995 ▸). Under irradiation, radical species with both strong oxidizing reducing power, such as hydroxyl radicals (OH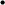) and hydrated electrons (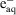), can be generated (Rehn & Jones, 2018 ▸). The significant decrease in the Cu^2+^ component (∼931 eV), together with the simultaneous increase in the Cu^+^ component (∼934 eV) over time, clearly indicates that beam-induced radiolysis produced reducing species capable of reducing Cu oxides. The recovery of the Cu^2+^ signal at a fresh position further corroborates that the reduction process is localized and induced by the X-ray beam.

In this context, assessing possible beam-induced damage before data acquisition, once the sample is in contact with the electrolyte, is a standard part of our experimental procedure. This effect is highly sample-dependent and is not always observed under our experimental conditions. When necessary, mitigation strategies are implemented, such as reducing the beam density by adjusting the beamline configuration (*e.g.* detuning the undulator harmonic or defocusing the beam), reducing beam exposure time by blocking the beam between spectra acquisition, or varying the irradiated position on the sample to distribute the exposure. Moreover, the use of a scavenger, such as iso­propanol, whenever compatible with the catalytic system under investigation, has been reported in the literature as an effective strategy to reduce the concentration of radiolytic products (Rehn & Jones, 2018 ▸).

Nevertheless, as reported in Fig. 4 ▸(*b*), a decrease in the Cu^2+^ signal intensity was also observed in a similar experiment without a clear appearance of the Cu^+^ feature around 934 eV. This indicates that, in this case, the decrease in the Cu^2+^ component is more plausibly associated with partial dissolution or detachment of the sample from the membrane than to beam-induced damage, as generally shown by post-measurement visual or optical microscope inspection of the membrane. To improve the stability of the Cu catalyst under reaction conditions, a coating layer of commonly used Nafion ionomer binder was applied. In fact, the stability of samples prepared on the membrane is the most critical aspect of these measurements and is often underestimated.

### 2.2. Cells

Here we present two electrochemical cells developed for soft X-ray absorption experiments under *operando* conditions: (1) a three-electrode electrochemical microfluidic cell (EC-cell); (2) a two-electrode cell specifically designed for charge and discharge experiments on battery materials (B-cell).

#### 2.2.1. EC-cell

In our previous work (Nappini *et al.*, 2021 ▸), we designed and developed an electrochemical microfluidic cell for *operando* measurements. Based on this prototype, we developed a new cell with improved performance with respect to the old one.

The working principle of the new EC-cell is similar to the previous one [for more details see Nappini *et al.* (2021 ▸)]. Briefly, a Si_3_N_4_ (or SiC) membrane separates the liquid from the vacuum allowing the cell to operate in vacuum conditions (1 × 10^−8^ mbar); two microfluidic channels allow complete electrolyte exchange when the cell is placed in a vacuum chamber and enable measurements under both convective and static conditions; the membrane, coated with a conductive layer of Ti/Au, is the working electrode on which the catalyst material is deposited, either *in situ* by electrodeposition (Nappini *et al.*, 2021 ▸) or *ex situ* by drop-casting (Cattelan *et al.*, 2025 ▸) and other deposition methods, such as atomic layer deposition, chemical vapor deposition, plasma-enhanced chemical vapor deposition, or sputtering (Di Costola *et al.*, 2025 ▸; Arnouts *et al.*, 2025 ▸).

The new cell has been optimized with several improvements: (1) a standard Ag/AgCl reference electrode has been added, (2) the electrode geometry has been modified, (3) the cell volume has been increased, and (4) the sealing of the cell has been improved, making the assembly of the Si_3_N_4_ membrane simpler, preventing its damage, and making it more reproducible and suitable even for non-expert users.

**Figure 5:**
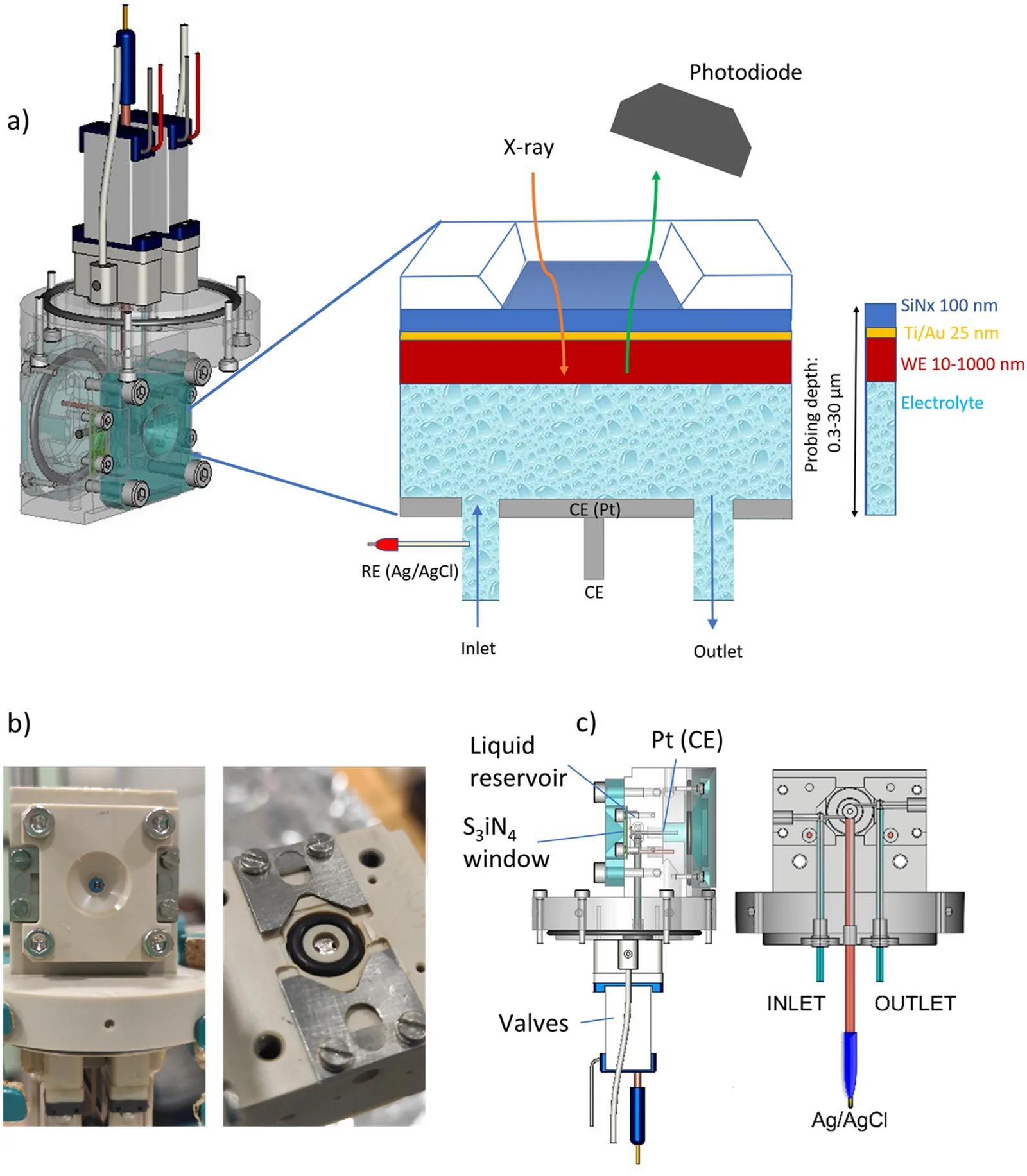
(*a*) Schematic drawing of the EC-cell (not to scale). (*b*) Photographs of the fully assembled EC-cell and in open configuration. (*c*) A 3D technical drawing of the cell design, along with a technical diagram showing the electrolyte pathways within the cell.

In the new configuration, a large Pt counter electrode covers nearly the entire bottom part of the cell (see Fig. 5 ▸) and is positioned in front of the WE with a similar size. This is important for achieving a more uniform current distribution, reducing potential geometry-dependent polarization effects, and enhancing the stability of the electrode and reaction kinetics.

For electrodeposition purposes, this configuration generates a more homogeneous electric field and current distribution across the working electrode surface, thereby minimizing edge effects. Consequently, both the local current density and the ionic flux in the electrolyte are more evenly distributed. Altogether, these factors contribute to a more uniform deposition rate and improved film homogeneity.

The reference electrode is a commercial miniaturized leakless saturated Ag/AgCl electrode (eDAQ) positioned in the inlet microfluidic channel to ensure good contact with the solution.

The electrochemical performance of the cell has been evaluated by a direct comparison between the electrochemical response obtained in our *in situ* EC-cell and in a standard beaker-type electrochemical cell under identical conditions (see Section S2).

The total volume of the cell, geometry and reservoir filling has been optimized to reduce bubble formation. The total volume of the cell is ∼20 µl. The cell is filled from the bottom, while the outlet is located at the top of the well, enabling complete filling and gas removal using a peristaltic pump (LEADFLUID, BT103S).

The cell is mounted on a long tube sealed by an NBR (Nitrile, Buna-N) O-ring, where the microvalves are located in air along with the flow tubes and the electrical wires. The tube is mounted on the manipulator that enables a precise alignment of the cell along the *x*, *y*, *z* and ϑ axes.

#### 2.2.2. Battery cell (B-cell)

A second cell has been specifically designed for battery materials. The development of high-energy-density rechargeable alkali-ion batteries is the main trend in the battery field, with two main approaches via increasing the capacity of the electrodes or using cathode materials with a higher redox potential. The development of 5 V Li-ion batteries is still challenging due to the intrinsic voltage limit of the cathodes, the electrolyte decomposition accompanied by gas evolution at charging potential above 4.6 V versus Li^+^/Li (Reuter *et al.*, 2024 ▸), the formation of a poor Li-ion conducting cathode–electrolyte interface (CEI), and the difficulties to reach the thermodynamic equilibrium at >5.0 V versus Li^+^/Li.

The main advantage of *operando* soft-XAS experiments is the ability to monitor the evolution of the oxidation states of redox-active materials under real operating conditions of a battery cell. This approach avoids voltage drop and self-discharge effects that occur after interrupting charge–discharge cycling, which can alter the oxidation states at a selected charging potential.

The concept of the cell, proposed in our work, is a simple static cell, based on a two-electrodes half-cell configuration, optimized for assembly in a glove box.

**Figure 6:**
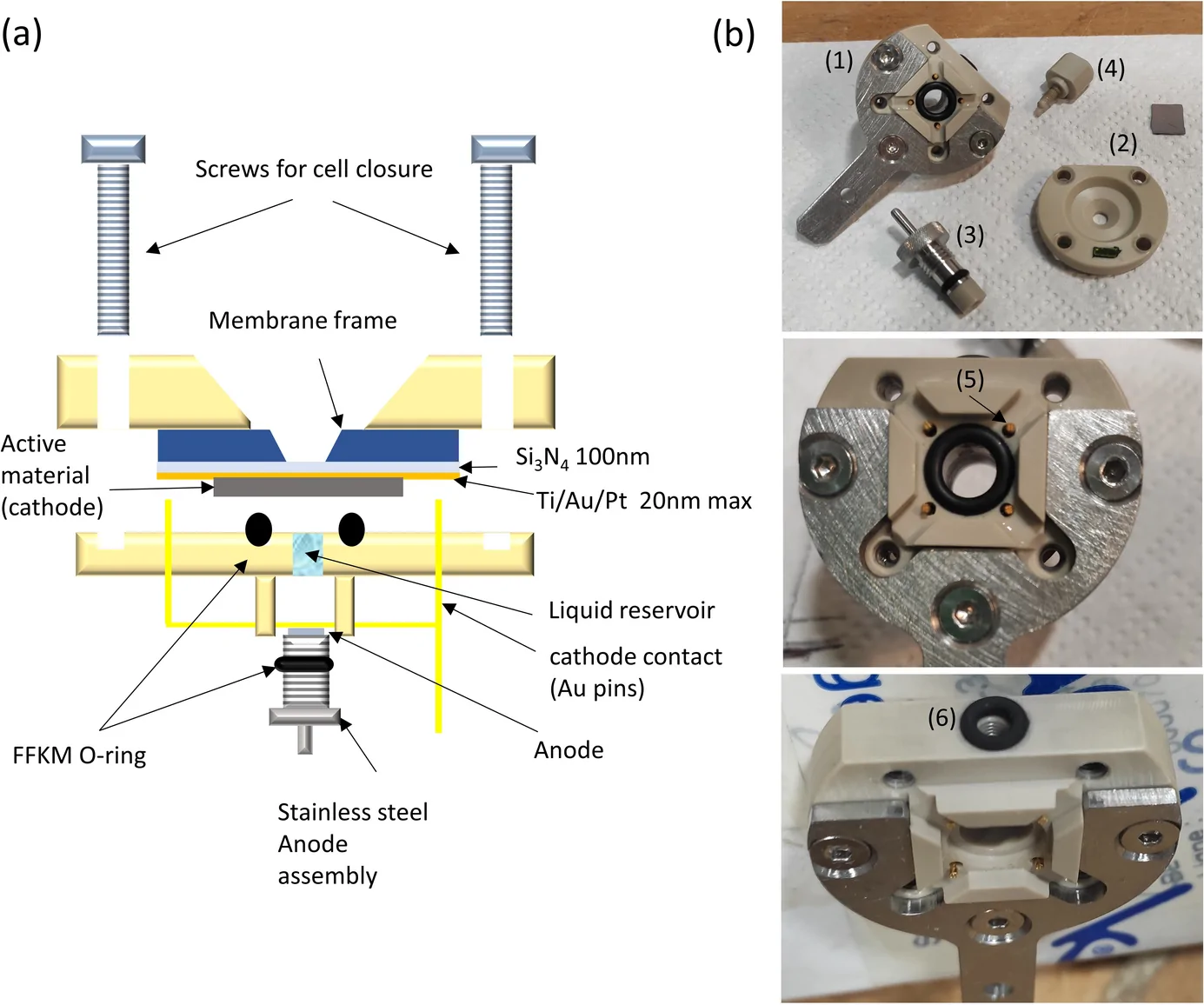
(*a*) Schematic drawing of the B-cell design (not to scale). (*b*) Photographs of the battery-cell construction parts: (1) central body in PEEK on a stainless steel support, (2) PEEK cap, (3) stainless steel anode assembly, (4) plug, (5) gold-plated pin contacts, (6) top opening.

The cell consists of four main components: (1) a central body made of polyether ether ketone (PEEK), mounted on a stainless steel support that enables precise positioning of the cell within the experimental chamber; (2) a PEEK cap that seals the liquid in the well using an X-ray-transparent membrane; (3) a stainless steel anode assembly; and (4) a plug that seals the liquid in the well (see Fig. 6 ▸).

The cell body is designed with a square housing unit on one side to hold the Si_3_N_4_ membrane, on which the Ti/Au current collector [Fig. 6 ▸(*b*)] and the thin-film active material (AM) is consequently deposited. The redox active material acts as the cathode (positive electrode). On the opposite side, a circular compartment houses the anode foil (negative electrode) secured by a dedicated PEEK hollow cap. The Si_3_N_4_/Ti/Au/AM sandwich structure is placed in the square part over an FFKM (perfluoro­elastometer) gasket which seals the liquid chamber. When the PEEK cap is screwed onto the cell body, the sandwich structure is mechanically pressed against the four small retractable gold-plated pin contacts, ensuring electrical connection. On the anode side, a metal plate is mounted on a stainless steel mushroom-shaped holder that is screwed to seal the rear compartment. Once the three parts are assembled, the electrolyte can be introduced through the top opening using a syringe. A small tube can then be connected to the same port to allow for gas release during electrochemical cycling (especially at high operating voltage), preventing pressure build up that could otherwise damage the Si_3_N_4_ membrane. Finally, the cell is sealed in a glovebox, transported to the BACH endstation, and inserted into the XAS measurement chamber [Fig. 3 ▸(*a*)].

### 2.3. Manipulator

A four-axis manipulator (CREATEC) was custom-adapted to hold the cells and to enable easy mounting of new cells specifically developed by users for particular experiments.

The manipulator is mounted at the bottom of the chamber (see Fig. 2 ▸). This configuration has been chosen to allow easy mounting of the EC-cell and to facilitate working conditions, given the height of the beamline.

A CF flanged-rod designed to host the B-cell can be mounted on the manipulator before inserting the B-cell and can be adapted upon request to host different cells.

The manipulator enables straightforward insertion of the supports and ensures easy mounting and handling of both the EC-cell and the B-cell. Importantly, the design is not limited to these specific configurations: user-developed cells can be accommodated through the same interface. In particular, the CF flanged rod originally designed for the battery cell can be readily adapted, upon request, to host different types of cells. This flexibility makes the setup compatible with a wide range of emerging designs, including 3D-printed cells, while preserving the key requirement of operation under UHV conditions.

Since all electrical connections and liquid flow lines are located on the air side, the system can be easily adapted to accommodate different cells provided by users for their experiments.

## 3. Performance

### 3.1. EC-cell

To demonstrate the performance and applicability of the developed EC-cell under realistic working conditions, two representative *in situ* XAS experiments are presented. These examples illustrate how the developed cell designs enable detailed *in situ* investigations of the solid–liquid interface and its dynamic structural and electronic modifications under electrochemical operating conditions.

Copper and iron nanoparticles (NPs) were selected as test materials due to their well established catalytic properties. Both metals are earth-abundant and cost-effective, making them ideal candidates for scalable electrochemical applications. Cu NPs exhibit excellent catalytic activity for reactions such as CO_2_ reduction (Yan *et al.*, 2024 ▸) and hydrogen evolution (Shi *et al.*, 2023 ▸), while Fe NPs are known for their role in oxygen reduction (Liu *et al.*, 2022 ▸) and environmental remediation processes (Chaudhari *et al.*, 2024 ▸). Their distinct electrochemical behaviors and ease of synthesis provide valuable benchmarks for assessing the performance, stability and deposition control of the new electrochemical setup. Studying Cu and Fe thus offers a robust foundation for optimizing experimental parameters before extending the system to more complex or precious catalytic materials.

A Cu-based catalyst was used as a benchmark system to monitor electrodeposition and redox behavior under alkaline conditions. The second example focuses on Fe-containing catalysts, representative of materials typically investigated for oxygen evolution and related redox reactions. Together, these case studies highlight the versatility of the cells for *in situ* and *operando* XAS investigations across different electrochemical environments and reaction conditions.

#### 3.1.1. *In situ* Cu NPs electrodeposition in acidic media

Electrodeposition is a widely employed synthesis method for the preparation of electrocatalytic materials, largely due to the unique combination of control, versatility and accessibility it offers. Through the precise adjustment of deposition parameters, this technique enables accurate tuning of the catalyst thickness, composition and morphology, allowing researchers to engineer surfaces ranging from single metals to alloys, metal oxides and even high-entropy alloys (Miao *et al.*, 2022 ▸).

In this work, the electrodeposition process of copper nanoparticles (Cu NPs) from an acidic electrolytic solution (10 m*M* CuSO_4_·5H_2_O + 10 m*M* Na_2_SO_4_/H_2_SO_4_) was *in situ* investigated using the EC-cell described above. Electrodeposition was performed galvanostatically using a two-electrode configuration by applying a constant current density between the working electrode and the Pt counter electrode. To determine the optimal deposition parameters, preliminary experiments were performed under *ex situ* conditions using the same EC-cell and configuration [see Section S3 and Napal Azcona (2025 ▸) for further details].

The *ex situ* XAS measurements [see Fig. S3(*b*)] of the synthesized Cu NPs reveal the formation of catalytic material composed of a mixture of Cu^0^, Cu^1+^ and Cu^2+^ oxidation states. The differences observed between the total electron yield (TEY) and total fluorescence yield (TFY) spectra underscore the strong susceptibility of copper to oxidation when exposed to air, which can significantly modify its intrinsic properties. This behavior emphasizes the necessity of *in situ* characterization to reliably capture the actual state of the catalytic material during electrodeposition.

**Figure 7:**
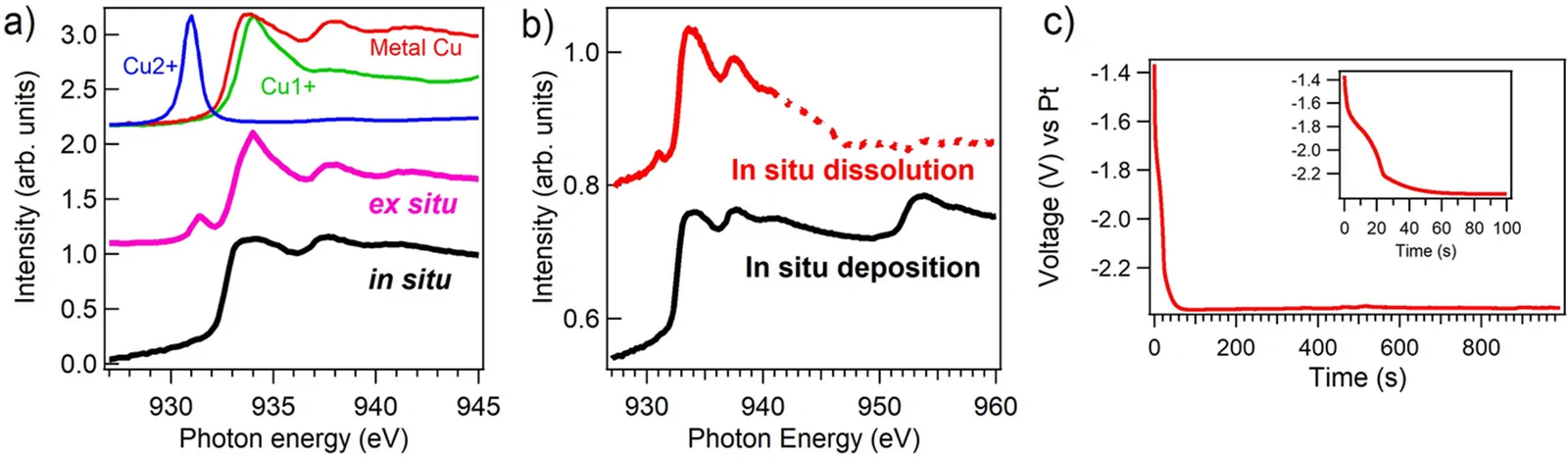
(*a*) Cu *L*_3_-edge TFY spectra for *ex situ* and *in situ* electrodeposited Cu nanoparticles. (*b*) Cu *L*_3,2_-edge spectra acquired under *in situ* conditions. The black curve shows galvanostatic electrodeposition, while the red curve corresponds to the spectrum acquired during deposition followed by current interruption; the solid red line is recorded under applied current, and the red dashed segment (940–960 eV) indicates the intensity decrease after switching off the current, consistent with progressive Cu dissolution. (*c*) Potential evolution over 1000 s during *in situ* electrodeposition under a cathodic current density of −1.3 mA cm^−2^. Insert: image of the first 100 s.

Fig. 7 ▸(*a*) shows the Cu *L*_3_-edge spectra measured in TFY for nanoparticles electrodeposited *ex situ* and *in situ* under the same galvanostatic conditions, along with the reference spectra of Cu^0^ metal, Cu^1+^ and Cu^2+^ oxides.

The *in situ* XAS measurements indicate that, under the applied electrodeposition conditions (*j* = −1.3 mA cm^−2^), the nanoparticles consist fully of metallic copper, as shown by the pronounced features at 933.8 eV and 938 eV. In contrast, following *ex situ* electrodeposition and subsequent exposure of the catalyst to air, a significant degree of oxidation is observed. The Cu^1+^ contribution at 934 eV increases significantly, together with a rise in the Cu^2+^ component at 931 eV. These results are evidence of the strong oxidation of copper when the material is removed from the electrochemical environment.

Moreover, the *in situ* XAS measurements recorded during copper electrodeposition reveal not only the formation of metallic copper but also the onset of its dissolution in the acidic electrolyte [Fig. 7 ▸(*b*)] when the applied bias was switched off.

During the *in situ* electrodeposition experiment, when the constant current density was applied, the potential changed around a value of approximately −2.35 V versus Pt [see Fig. 7 ▸(*c*)]. In this condition, solvated Cu^2+^ ions migrated toward the negatively polarized working electrode surface (Ti/Au Si_3_N_4_ membrane), where the electron transfer process occurred and metallic copper is formed, as reflected by the Cu *L*_3,2_-edge black spectrum in Fig. 7 ▸(*b*). However, if the bias is switched off before the Cu *L*_3,2_-edge acquisition is completed, the system returns to open-circuit potential (OCP, ∼−0.1 V versus Pt), where the freshly deposited copper begins to corrode, leading to the formation of Cu^2+^ species that subsequently re-dissolve into the electrolyte, in agreement with the Pourbaix diagram (Beverskog & Puigdomenech, 1997 ▸). This process is reflected by the progressive decrease in spectra intensity above ∼940 eV during the transient increase in potential toward OCP stabilization, as shown by the red dashed segment of the spectrum in Fig. 7 ▸(*b*).

#### 3.1.2. Cu NPs redox process in alkaline media

Understanding the redox behavior of copper in alkaline media, such as KOH, is crucial for its rational use as an electrocatalyst. In this work, the oxidation and reduction transitions that copper undergoes upon applying potential in an alkaline environment were studied *in situ* by measuring the Cu *L*_3,2_-edge spectra at selected redox potentials.

Nevertheless, since the *in situ* XAS experiments conducted during copper electrodeposition revealed the rapid dissolution of copper in acidic medium once the applied potential is removed, the Cu NP sample for redox study was prepared *ex situ*. In this approach, the sample is extracted from the acidic electrolyte while the deposition potential is still applied, thereby minimizing copper dissolution and preserving the integrity of the deposited nanoparticles. However, due to unavoidable air exposure during transfer, a certain degree of copper oxidation is expected.

**Figure 8:**
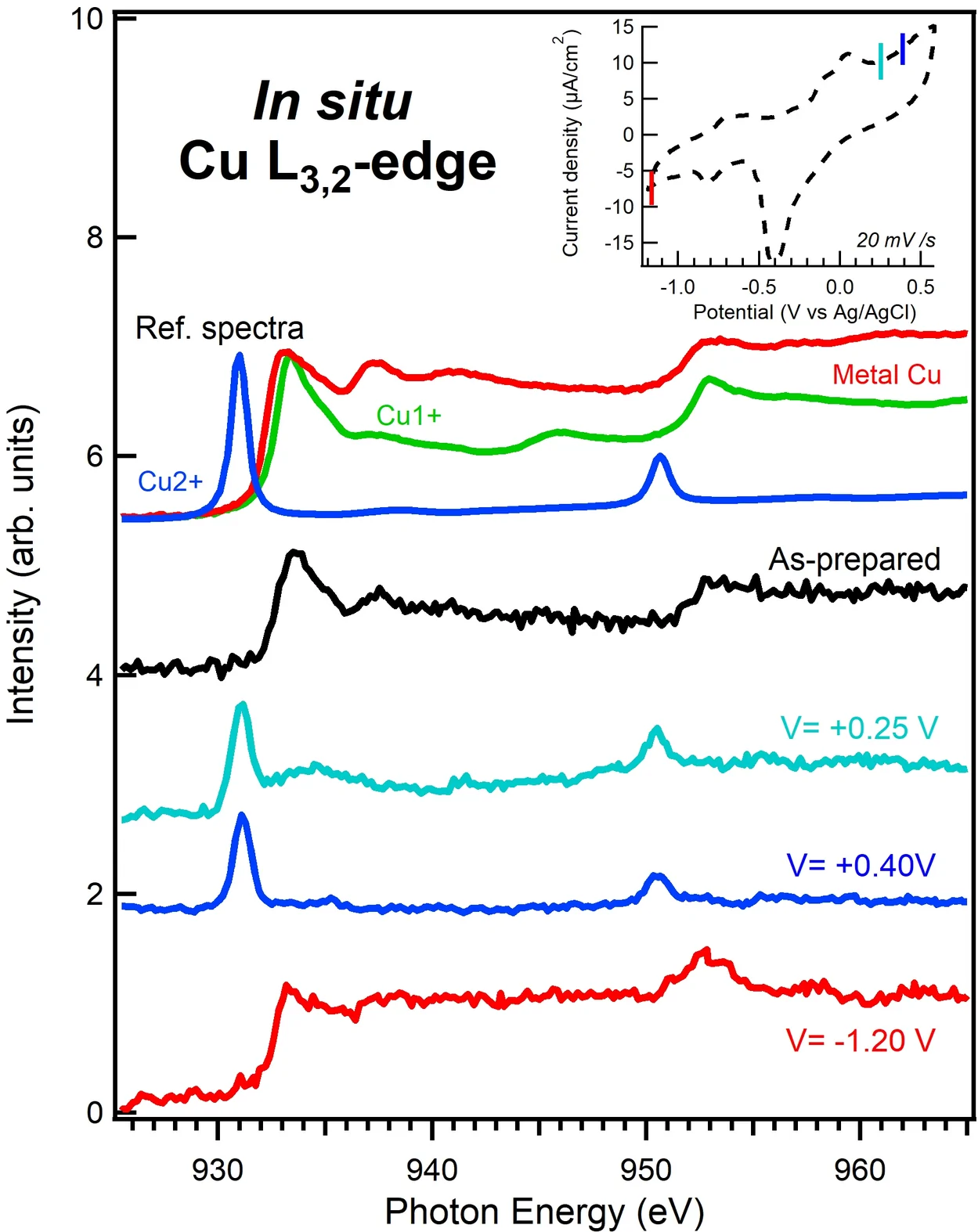
*In situ* Cu *L*_3,2_-edge XAS spectra acquired at selected redox potentials. The spectra of reference sample, metallic copper foil, Cu_2_O and CuO powders are shown at the top. The inset displays the cyclic voltammetry curve measured in 0.1 *M* KOH using the EC-cell; vertical colored ticks indicate the potentials applied during spectral acquisition. The applied potentials indicate in the figure are reported versus saturated Ag/AgCl reference electrode.

The inset panel in Fig. 8 ▸ shows the *in situ* cyclic voltammetry (CV) recorded on the copper NPs in the EC-cell in 0.1 *M* KOH medium. The CV curve shows a clear ohmic resistance compared with similar systems [see, for example, Garcia-Diez *et al.* (2025*b* ▸)]; however, the main redox features appear at comparable potentials, confirming that the underlying electrochemical processes remain consistent with previous reports (see Section S2 for a detailed discussion). Three different potentials were chosen to monitor the oxidation (*V* = 0.25 and 0.4 V versus Ag/AgCl) and the reduction (*V* = −1.2 V versus Ag/AgCl) behaviors of the catalytic material.

The Cu *L*_3,2_-edge spectrum of the initial Cu nanoparticles under OCP conditions (black spectrum in Fig. 8 ▸) exhibits a dominant feature at ∼934 eV, characteristic of the Cu^1+^ oxidation state, together with a pronounced peak at ∼937.8 eV indicative of a more metallic Cu component, as expected from previous experiments. Upon applying an anodic potential of 0.25 V versus Ag/AgCl, a peak at 931 eV related to the bivalent copper appears, while the contribution of oxidized copper in the form of Cu_2_O decreases markedly. Complete oxidation of Cu nanostructures is achieved at 0.4 V versus Ag/AgCl, as shown by the dis­appearance of the 934 eV feature. These results indicate that, although CV clearly resolves the oxidation peaks, the bulk of the material does not undergo complete oxidation precisely at that specific potential. It is also plausible that complete oxidation could be achieved at lower potentials (*e.g.* 0.25 V), if the bias is held for a sufficiently long duration. The reduction of Cu^2+^ nanoparticles to metallic Cu was carried out by applying a cathodic potential of −1.2 V versus Ag/AgCl, enabling continuous monitoring of the oxidation state of the material during a full CV cycle. Overall, these findings highlight the capability of the EC-cell to track dynamic changes in the oxidation state under electrochemical control.

#### 3.1.3. Fe_*x*_O_*y*_ NPs: electrodeposition, oxidation and reduction in water

**Figure 9:**
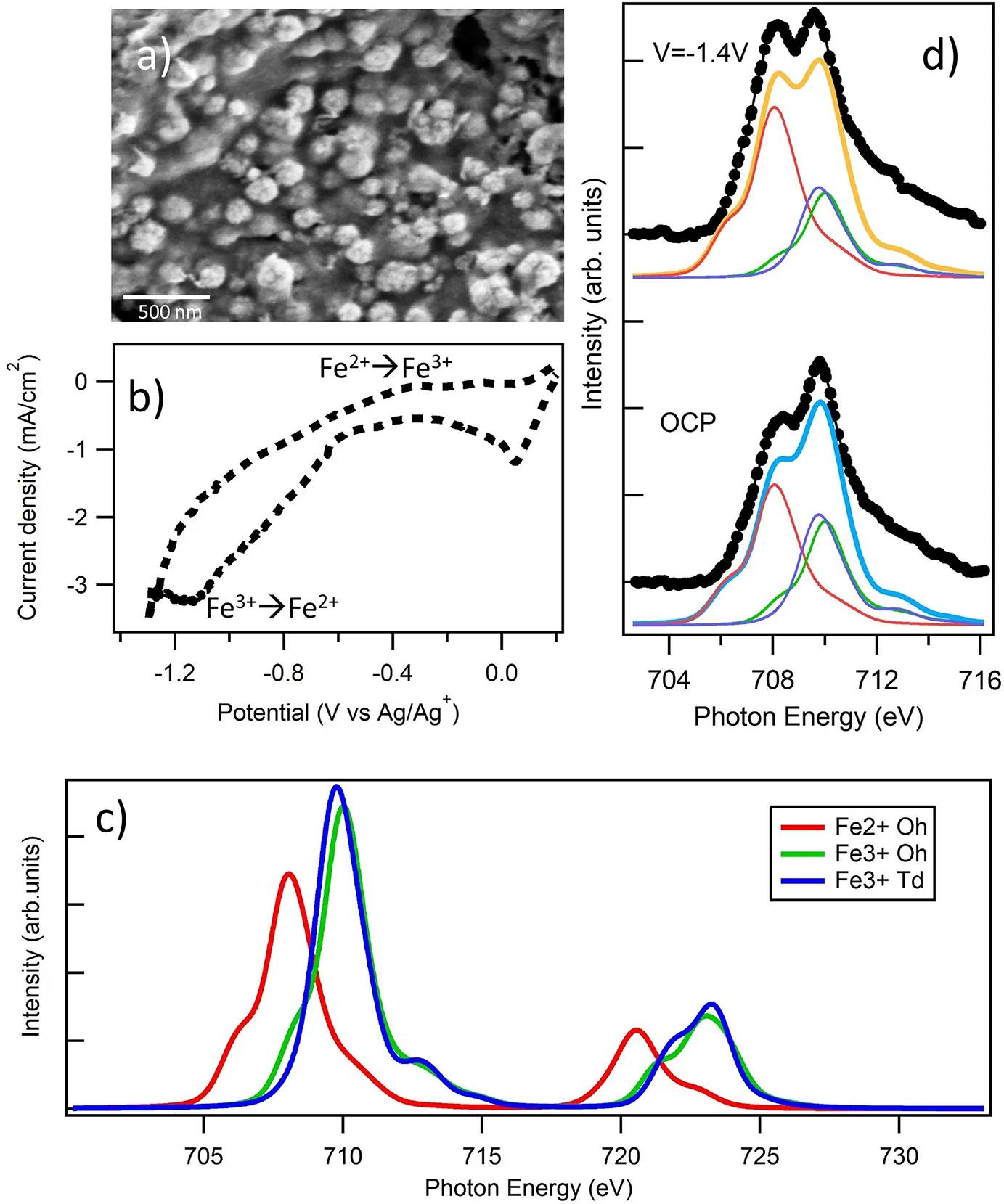
(*a*) SEM image of electrodeposited Fe_*x*_O_*y*_ NPs. (*b*) Cyclic voltammetry curve measured in water on Fe_*x*_O_*y*_ NPs. (*c*) Fe *L*_3,2_-edge XAS calculations by Cowan’s multiplet program for Fe^2+^ Oh (red curve), Fe^3+^ Oh (green curve) and Fe^3+^ Td (blue curve); (*d*) *operando* Fe *L*_3_-edge spectra for pristine Fe_*x*_O_*y*_ NPs (bottom, black) and for the reduced material under an applied voltage of −1.4 V (top, black). The corresponding calculated curves are displayed beneath each spectrum, together with the weighted sums that best reproduce the experimental data.

*In situ* electrodeposition of iron oxide nanoparticles (Fe_*x*_O_*y*_ NPs) was achieved from an aqueous solution of Fe(NO_3_)_3_ (0.1 *M*) with the addition of 2 wt% ethyl­ene glycol under a constant current *j* = −3.3 mA cm^−2^ for 60 s. Fig. 9 ▸(*a*) shows an SEM image of the obtained layer consisting of NPs with an average dimension of around 150 nm.

Magnetite (Fe_3_O_4_) was formed through a cathodic precipitation–conversion mechanism. At the cathode, Fe^3+^ ions from the 0.1 *M* precursor are partially reduced to Fe^2+^, while nitrate and water reduction generate hydroxide ions and raise the local pH. Their coexistence, sustained by the continuous cathodic generation of Fe^2+^, enables an *in situ* solid-state conversion in which hydroxide ions dehydrate to form Fe_3_O_4_. Ethyl­ene glycol aids this process by weakly complexing Fe^3+^ and stabilizing Fe^2+^, thereby suppressing over-oxidation and promoting the formation of the mixed-valence phase required for magnetite.

XAS at the Fe *L*_3,2_-edge reveals the characteristic multiplet features of Fe_3_O_4_ (Chang *et al.*, 2016 ▸). Fe_3_O_4_ has an inverse spinel-type structure with half of the Fe^3+^ ions tetrahedrally coordinated (Td), whereas the remaining Fe^3+^ ions, as well as the Fe^2+^ ions, occupy octahedrally coordinated sites (Oh).

Fig. 9 ▸(*c*) shows the calculated XAS Fe *L*_3,2_-edge spectra for the three different sites Fe^2+^ *d*^6^ Oh, Fe^3+^ *d*^5^ Td, and Fe^3+^*d*^5^ Oh following the method of van der Laan & Thole (1991 ▸) using Cowan’s multiplet program (https://anorg.chem.uu.nl/CTM4XAS/software.html) based on Hartree–Fock code with relativistic correction. Interatomic screening and mixing were taken into account by reducing the *d*–*d* and *p*–*d* Slater integrals with scaling factors of 0.7 and 0.8, respectively. For the Fe^3+^ Oh and Td sites a crystal field of 10Dq = 1.25 eV was used, for the Fe^2+^ Td site a crystal field of 0.5 eV was used in agreement with Arenholz *et al.* (2006 ▸). The calculated results were broadened by a Lorentzian of 0.2 eV to account for intrinsic linewidth broadening and a Gaussian of 0.5 eV for the instrumental broadening.

From the comparison of the calculated and measured (after background subtraction) Fe *L*_3_-edge [see Fig. 9 ▸(*d*)], we estimate around 40% Fe^2+^ Oh, 30% Fe^3+^ Td, and 30% Fe^3+^ Oh, suggesting a reduction of Fe^3+^ ions to Fe^2+^ coordination.

After deposition, Milli-Q water was first fluxed in the cell and the cyclic voltammetry curve reported in Fig. 9 ▸(*b*) was measured. The CV reveals the Fe reduction peak at −1.2 V (versus Ag/AgCl) while the oxidation peak is not clearly visible; the pronounced peak around 0 V has been ascribed to O_2_ reduction reaction, due to the presence of O_2_ contamination in the solution.

To demonstrate the feasibility of performing *in situ* reactions on the electrodeposited material, we applied a potential of −1.4 V (versus Ag/AgCl) and measured the Fe *L*-edge spectrum under *operando* conditions. As shown in Fig. 9 ▸(*d*), the Fe *L*_3_-edge shows a clear increase of the feature located around 708.2 eV ascribed to a further reduction of Fe^3+^ to Fe^2+^. Referring to the multiplet calculations as shown in Fig. 9 ▸(*c*), we obtained an increase of Fe^2+^ in octahedral sites from 40% to around 56%.

### 3.2. Battery cell (B-cell)

LiCoPO_4_ (LCP) olivine structure thin-film cathode material (redox potential of −4.8 V versus Li^+^/Li) was used to study the evolution of the oxidation state of cobalt and the lattice oxygen, as well as chemical composition at the CEI by using the developed setup for *operando* XAS. Thin-film cathode materials prepared under UHV provide well defined model systems as they are contamination free, and do not contain conductive carbon or binders, which can be involved in unwanted electrochemical reactions above 4.6 V.

**Figure 10:**
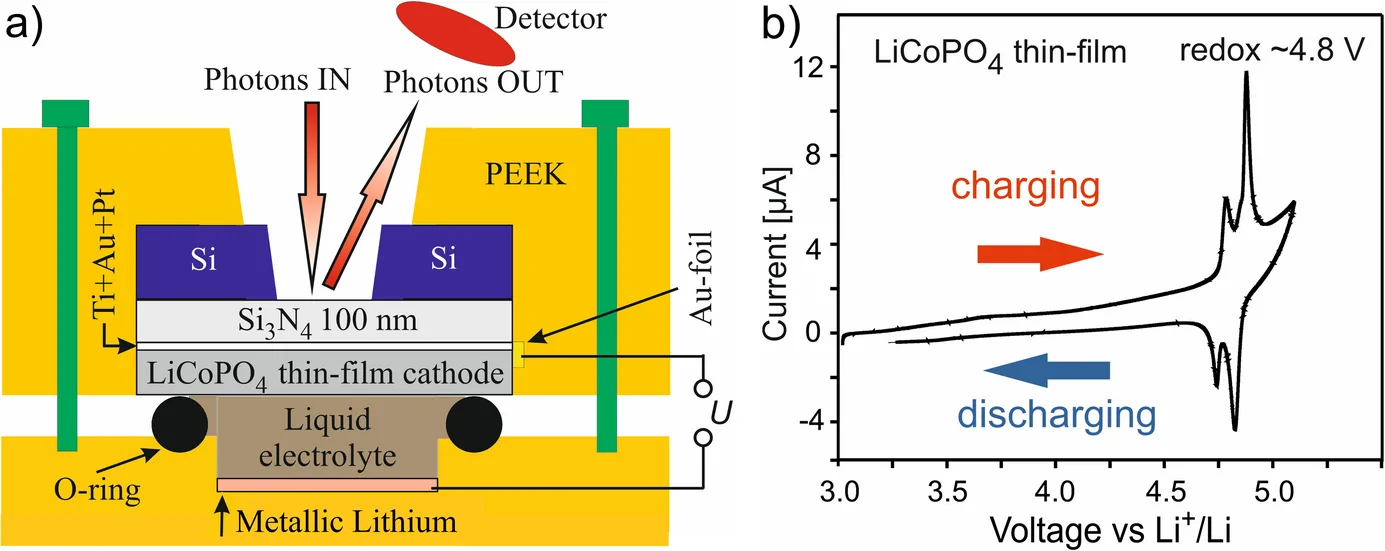
(*a*) Schematic drawing of the B-cell (not to scale) with LCP thin-film cathode, 1 *M* LiPF_6_ in 4:1 *w*/*w* DMC:FEC with 0.2 wt% TMB electrolyte solution and lithium anode, assembled for *operando* XAS measurements. (*b*) A typical cyclic voltammogram of the battery cell with LCP thin-film cathode (400 nm thick) deposited on Pt foil as the current collector. A lithium metal foil was used as the anode.

We deposited LCP thin-films (∼130 nm) on 100 nm-thick Si_3_N_4_ membranes. Preliminary, Ti (5 nm) and Au (15 nm) and Pt (3 nm) layers, as current collector, were deposited onto the membrane surface [Fig. 10 ▸(*a*)]. The LCP deposition was performed under argon (10^−2^ mbar) followed by thin-film annealing in synthetic air (99.99% purity, 80% N_2_ and 20% O_2_) at around 670°C (Cherkashinin *et al.*, 2017 ▸; Cherkashinin *et al.*, 2020 ▸). The B-cell was assembled in an Ar glove box (MBraun LabStar 50, H_2_O and O_2_ below 10 p.p.m.) as depicted in the schematic drawing in Fig. 10 ▸(*a*). The membrane was used to seal the cell with the liquid electrolyte 1 *M* LiPF_6_ in 4:1 *w*/*w* di­methyl carbonate (DMC): fluorinated ethyl­ene carbonate (FEC) with 0.2 wt% tri­methyl­boroxine (TMB).

**Figure 11:**
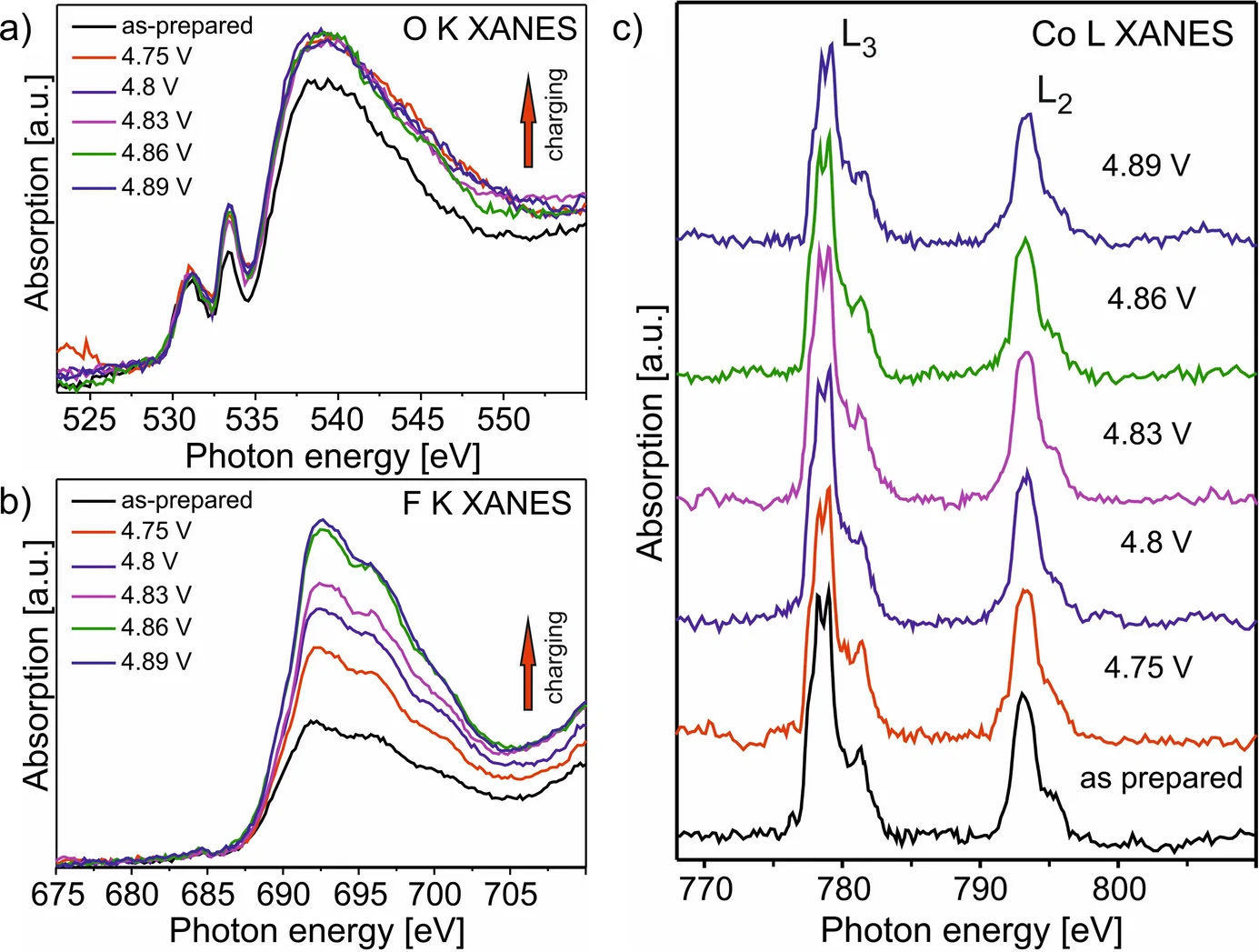
*Operando* XAS measurements: evolution of (*a*) O *K*-edge spectra, (*b*) F *K*-edge spectra, and (*c*) Co *L*_3,2_-edge spectra of the LCP thin-film cathode and the LCP/electrolyte interface upon charging LCP battery.

Fig. 10 ▸(*b*) shows a CV of the battery cell with LCP thin-film cathode grown at the same deposition parameters as for *operando* XAS. The B-cell was charged from OCP to 5.1 V and subsequently discharged to 3.0 V at a scan rate of 0.1 mV s^−1^. At charging voltages of 4.75, 4.80, 4.83 and 4.89 V, a constant voltage was held for one hour to promote maximal Li-ion extraction, after which XAS measurements were performed. Fig. 11 ▸ shows O *K*-, Co *L*_3,2_- and F *K*-edge XAS spectra of as-prepared LCP thin-film cathode material and their evolution versus charging states in the 4.75–4.89 V range under *operando* conditions, measured in TFY mode. The Co *L*_3,2_-edge spectra did not show any significant changes during the battery charging process. Instead, an increase of the cathode/electrolyte interface thickness, as a function of the applied voltage and the measurement time, is manifested by an intensity increase of the F *K*-edge, associated with LiPF_6_ salt and FEC solvent and its decomposition. Such a thick interface can block Li^+^ transport across the CEI, thereby hindering Co^2+^ ↔ Co^3+^ redox process. In addition, LCP has very low ionic and electronic conductivity. As a result, Li^+^ extraction and electron transport are kinetically hindered, and the oxidation reaction is expected to be largely confined to the near-surface region (*e.g.* close to the current collector) rather than proceeding uniformly through the bulk. Since XAS measured in TFY mode is relatively bulk-sensitive, even if the cyclic voltammogram shows an oxidation feature, the corresponding changes in the bulk may be too small to be clearly detected in TFY. By contrast, variations at the F *K*-edge are primarily associated with the formation and evolution of the CEI, making the fluorine signal a more direct probe of interphase growth and composition changes.

## 4. Conclusions

A new endstation fully dedicated to soft X-ray absorption spectroscopy in liquid environments has been successfully developed and commissioned on one branch of the BACH beamline, one of the CNR beamlines at the Elettra Synchrotron radiation facility (Italy). It has been specifically designed to enable *in situ* and *operando* investigations of solid–liquid interfaces under realistic working conditions, while preserving the stringent vacuum requirements of soft X-ray spectroscopy.

The modular design of the endstation allows the accommodation of different cells, significantly expanding the experimental capabilities of the beamline. Two dedicated electrochemical cells have been implemented and validated: a microfluidic cell for interfacial electrochemistry, and a static battery cell optimized for *operando* studies of high-voltage electrode materials. The upgraded microfluidic electrochemical cell incorporates several key improvements over the previous prototype, including a standardized Ag/AgCl reference electrode, an optimized electrode geometry for improved current distribution, increased electrolyte volume, and a more robust and user-friendly membrane sealing system.

The performance of the microfluidic electrochemical cell has been demonstrated through representative *in situ* soft XAS experiments on copper and iron nanoparticle systems. These case studies highlight the capability of the setup to track dynamic changes in oxidation state, phase composition and redox reversibility under electrochemical control, and to directly observe processes such as metal deposition, oxidation, dissolution and reduction. In particular, the comparison between *in situ* and *ex situ* measurements clearly underlines the importance of *operando* characterization for capturing the true chemical state of highly reactive materials.

The battery-dedicated cell enables *operando* soft XAS studies of electrode materials under realistic charging conditions. Its application to LCP thin-film cathodes demonstrates the potential of the setup to probe both transition-metal redox chemistry and cathode–electrolyte interphase evolution at high voltages. The observed growth of fluorine-containing interphases at increasing charging voltages highlights the sensitivity of soft XAS to interfacial phenomena that critically affect battery performance and stability, and underscores the complementarity between electrochemical measurements and element-selective spectroscopic probes.

Overall, the new LIQUID endstation at BACH represents a significant advancement for soft X-ray*operando* spectro-electrochemistry, providing a flexible and powerful platform for studying electrochemical, catalytic and energy-related materials in liquid environments. The demonstrated versatility of the setup, combined with its user-oriented design, opens new opportunities for investigating complex interfacial processes relevant to catalysis, corrosion, electrosynthesis and next-generation battery technologies.

## 5. Related literature

The following references, not cited in the main body of the paper, have been cited in the supporting information: D’Amario *et al.* (2025 ▸); Funsten *et al.* (1997 ▸); Jiang *et al.* (2013 ▸).

## Supplementary Material

Photon flux calculation and details on the synthesis of copper catalyst by electrodeposition. DOI: 10.1107/S1600577526005825/lin5003sup1.pdf

## Data Availability

The data that support the findings of this study are available from the corresponding author on request.
