# Supplementary material for: The LIQUID endstation for in situ spectro-electrochemical studies on energy materials @ BACH beamline
Source: J Synchrotron Radiat. 2026 Jul 15;33(Pt 5):1542–53. doi: 10.1107/S1600577526005825 (PMC13549344; doi:10.1107/S1600577526005825)
Supplement: Supplementary file 1 [file s-33-01542-sup1.pdf]

JOURNAL OF SYNCHROTRON RADIATION

Volume 33 (2026)

Supporting information for article:

The LIQUID endstation for *in situ* spectro-electrochemical studies on energy materials @ BACH beamline

I. Napal Azcona, F. Salvador, Z. Liang, G. Cherkashinin, M. Sosič, D. Benedetti, E. Betz-Güttner, S. Dal Zilio, A. Fondacaro, F. Bondino, I. Píš, M. Lazzarino, S. Nappini and E. Magnano

S1. Photon flux calculation

The photon flux (φ) was calculated as follows:

$$\varphi = \frac{P}{Eph}$$

where *P* is the power and *Eph* is the photon energy.

The power for the photodiode can be calculated as the current measured on the AXUV photodiode divided by the responsivity (*R*).

$$P = \frac{I}{R}$$

The responsivity is proportional to the number of electron hole pairs generated in the AXUV photodiode. As the photodiode is exposed to electrons and ions, electron-hole pairs (carriers) are created; the electric field in the p/n junction separates the charges and drives the current in the external circuit. The silicon electron-hole creation energy is found to be 3.71 eV in silicon (Funsten et al., 1997). For photons, this value can be used to calculate the ideal responsivity in a lossless system, R = 1/3.71 electron charge/eV = 0.27 C/J = 0.27 A/W.

The photon flux value reported in the main text is the normalized photon flux, where the measured photon flux was normalized to the slits opening of 50 microns and to the ring current of 300 mA.

**Photon energy (eV)**	**Grating**	**Undulator**	**Entrance slit opening (µm)**	**Exit slit opening (µm)**	**Ring energy (GeV)**	**Ring current (mA)**	**Flux (ph/s)**	**Normalized flux**
177	SG1	LE	30	20	2	249	2.00E+11	1.00E+12
177	SG2	HE	30	20	2	249	1.08E+11	5.42E+11
400	SG2	HE	30	20	2	249	1.08E+11	5.42E+11
650	SG3	HE	30	20	2	249	3.40E+10	1.71E+11
970	SG3	HE	40	40	2	309	1.33E+11	2.02E+11

S2. Electrochemical response of in situ EC-cell

To demonstrate the reliable electrochemical performance of the EC-cell, cyclic voltammetry measurements (CV) and electrochemical impedance spectroscopy (EIS) measurements were performed to directly compare the electrochemical response obtained in our *in situ* EC-cell with that of a standard beaker-type electrochemical cell under identical conditions (Cu foil as WE and 0.1 M KOH electrolyte).

As shown in Figure S1 a), both configurations display the same characteristic voltammetric features associated with Cu redox processes in alkaline media. In particular, the positions of the main cathodic and anodic features are comparable, indicating that the fundamental electrochemical behaviour of the Cu electrode is preserved in the *in situ* EC-cell.

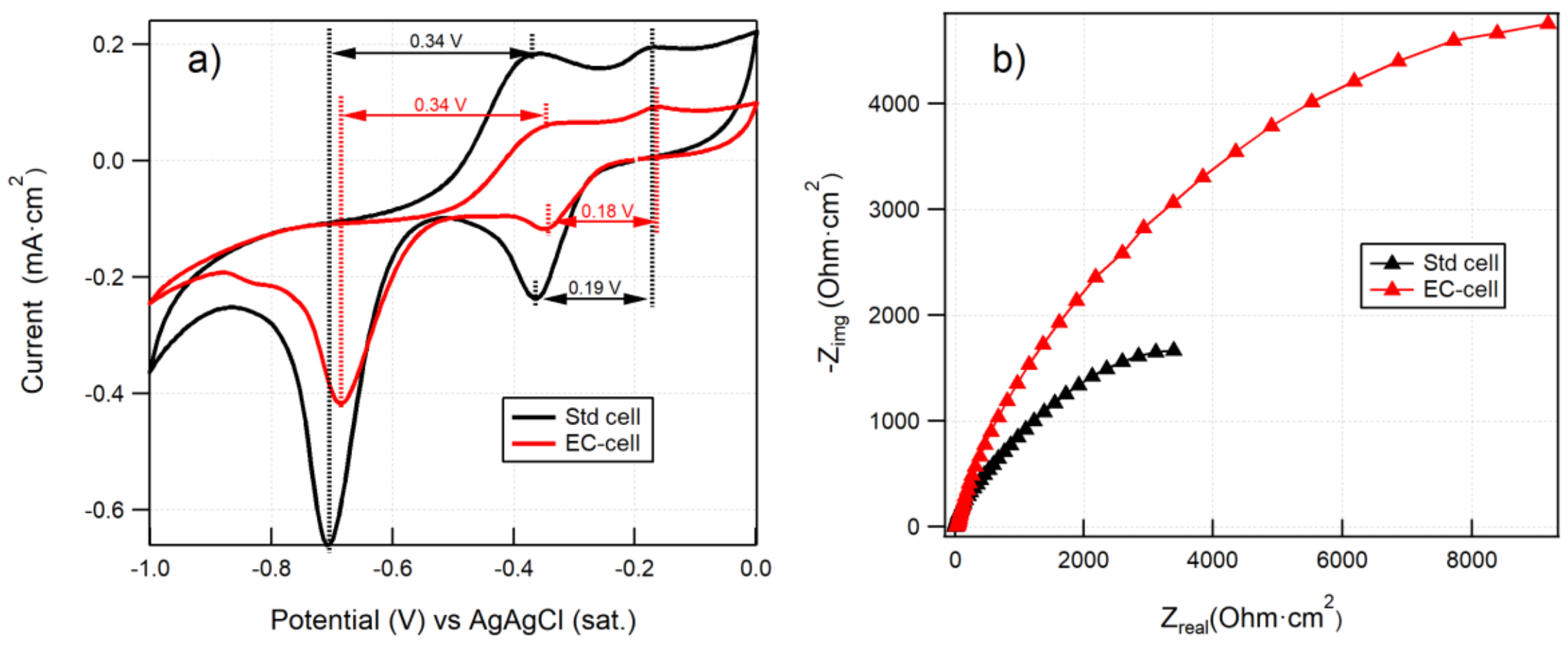

Figure S1 a) Comparison of CVs curves and b) Nyquist plots measured on Cu foil in the *in situ* EC cell (red) and in a standard beaker-type electrochemical cell (black).

However, clear differences are observed in the shape and intensity of the voltammetric response. In the standard cell, the peaks are slightly sharper and higher in current, while in the EC-cell they appear broader and with lower current density. This behaviour is consistent with the more confined geometry of the *in situ* cell, which contains a significantly smaller electrolyte volume (~20 µL) to ensure compatibility with the UHV environment and limiting the risk in case of membrane rupture. In contrast to the beaker configuration, this restricted volume limits the charge transfer.

To further investigate this aspect, EIS measurements were done. The Nyquist plots shown in Figure S1 b) clearly show that the EC-cell exhibits a higher impedance over the entire frequency range. The high-frequency intercept, associated with the uncompensated resistance (Rs), is slightly increased in the EC-cell, while a much larger divergence is observed at intermediate and low frequencies.

This higher Rs is consistent with the larger peak separation and potential shifts observed in the CVs. Moreover, these results indicate that the increased resistance observed in the CV response does not arise solely from a higher ohmic resistance but is predominantly related to transport and geometrical effects. The highly confined electrolyte volume (~20 µL) of the EC-cell may promote concentration gradients and limits mass transport, increasing the overall resistance of the cell.

Additionally, the configuration of the reference electrode plays a critical role. In the *in situ* EC-cell, the reference electrode is located in a separate channel, a design choice intended to prevent contamination or interference from species generated at the WE, thereby preserving the stability and reliability of the reference potential. This separation introduces an additional uncompensated resistance, which contributes to both the apparent ohmic drop in CV and the increased impedance observed in EIS.

Moreover, new cell designs are currently being developed to improve and overcome these limitations, particularly by optimizing the electrolyte volume, by increasing the flow rate, and enhancing reference electrode positioning (D'Amario, n.d.).

Importantly, despite these differences, the main redox features appear at comparable potential, confirming that the underlying electrochemical processes remain consistent with previous reports. This indicates that the electrochemical conditions achieved in the EC-cell can be considered reproducible with respect to conventional *ex situ* measurements, enabling a direct comparison between both configurations.

Overall, the EC-cell preserves the intrinsic electrochemical behaviour of the catalyst, while the observed differences arise from well-understood geometrical and transport limitations inherent to the small cell configuration. Consequently, the EC-cell provides a reliable platform to investigate electrocatalytic materials under conditions that closely resemble realistic operating environments, while maintaining compatibility with *in situ* XAS characterization technique in UHV environment.

S3. Synthesis of copper catalyst by electrodeposition

The electrodeposition of copper nanoparticles (Cu NPs) from an acidic electrolyte (10 mM $CuSO_4 \cdot 5H_2O$ + 10 mM Na_2SO_4/H_2SO_4) was initially carried out under *ex situ* conditions to identify the optimal deposition parameters—namely the applied potential and the deposition time—required to obtain uniformly distributed, small nanoparticles. (Napal Azcona, 2025). Figure S2 a) shows the SEM image of the Cu NPs electrodeposited on a silicon wafer coated by a thin layer of Ti/Au under galvanostatic conditions, using an applied current density of -1.3 mA/cm^2 for 40 seconds. The electronic structure of the obtained sample was first characterized *ex situ* by X-ray Absorption Spectroscopy (XAS). Figure S1 b) shows the Cu L_3-edge XAS spectra acquired in Total Electron Yield

(TEY) and Total Fluorescence Yield (TFY) modes. The TEY mode, which measures the drain current from the sample, provides surface-sensitive information, while the TFY mode, collected using a microchannel plate (MCP) detector, probes deeper into the material. The combination of these two detection modes enables a comprehensive analysis of both the surface and bulk electronic properties of the Cu nanoparticles.

For comparison, the reference Cu $L_{3,2}$-edge XAS spectra of Cu^0, Cu^{1+}, and Cu^{2+} measured on a Cu metal plate, and on Cu_2O and on CuO powders, respectively are also reported. The Cu $L_{3,2}$-edge of Cu_2O displays a main absorption resonant peak at around 934 eV attributed to $3d^{10}$ shell, along with less intense features at higher photon energy. In contrast, the CuO spectrum exhibits a strong resonant peak at 931 eV, corresponding to $3d^9$ electronic configuration (Jiang et al., 2013), accompanied by satellite structure at higher energy. In the case of metallic Cu, the spectrum shows three peaks located at 933.8, 938 and 941.4 eV, respectively.

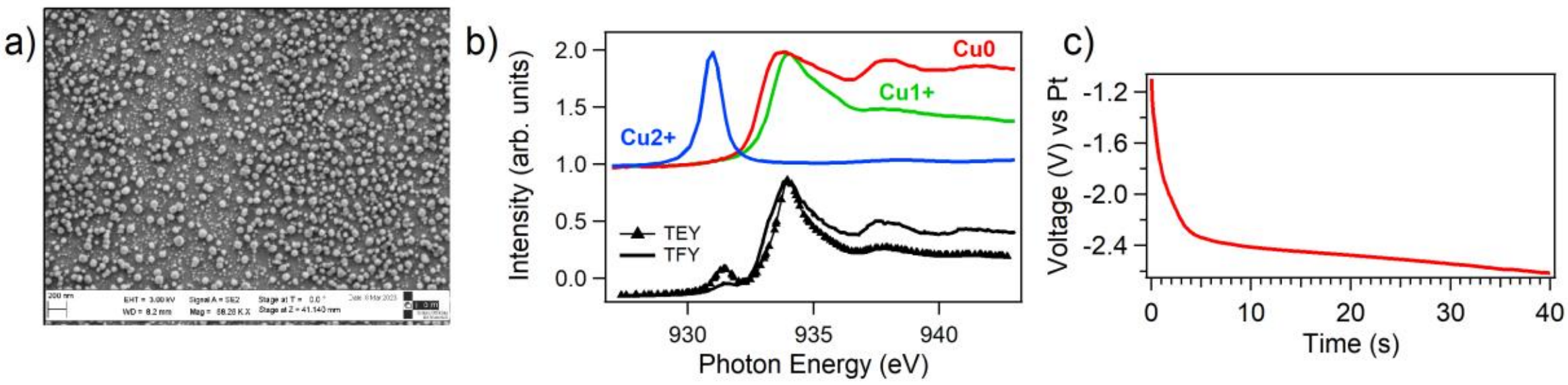

Figure S2 a) SEM image of galvanostatically electrodeposited Cu NPs on Ti/Au coated silicon wafer. b) *ex-situ* Cu L_3-edge XAS of pristine Cu NPs in TEY and TFY mode, shown together with reference spectra for metallic Cu, Cu_2O, and CuO. c) Potential evolution during 40 seconds electrodeposition time applying a cathodic current density of -1.3 mA/cm^2.

The XAS measurements reveal clear differences between the spectra acquired in TEY and TFY modes. From the TFY spectrum, we infer that the bulk of the material is predominantly composed of metallic copper, as indicated by the pronounced feature at ~937.8 eV, which is characteristic of Cu^0, although contributions of CuO and Cu_2O are also present. In contrast, the TEY spectrum shows a higher degree of surface oxidation, mainly in the form of Cu_2O. Moreover, the presence of the peak at ~931 eV evidences the contribution of CuO, while the reduced intensity of the 937.8 eV feature indicates a lower fraction of metallic Cu at the surface.

References

D'Amario, L., n.d. Soft X-ray Operando Spectroscopy for Gas-Evolving Electrocatalysis at 100 mA/cm2.

Funsten, H.O., Suszcynsky, D.M., Ritzau, S.M., Korde, R., 1997. Response of 100% internal quantum efficiency silicon photodiodes to 200 eV-40 keV electrons. IEEE Transactions on Nuclear Science 44, 2561–2565. https://doi.org/10.1109/23.650863

Jiang, P., Prendergast, D., Borondics, F., Porsgaard, S., Giovanetti, L., Pach, E., Newberg, J., Bluhm, H., Besenbacher, F., Salmeron, M., 2013. Experimental and theoretical investigation of the electronic structure of Cu_2O and CuO thin films on Cu(110) using x-ray photoelectron and absorption spectroscopy. The Journal of Chemical Physics 138, 024704. https://doi.org/10.1063/1.4773583

Napal Azcona, I., 2025. Spettroscopie a raggi X per un'economia basata sull'idrogeno.
